# The Nordic back pain subpopulation program: predicting outcome among chiropractic patients in Finland

**DOI:** 10.1186/1746-1340-16-13

**Published:** 2008-11-07

**Authors:** Stefan Malmqvist, Charlotte Leboeuf-Yde, Tuomo Ahola, Olli Andersson, Kristian Ekström, Harri Pekkarinen, Markku Turpeinen, Niels Wedderkopp

**Affiliations:** 1 The Faculty of Social Sciences, University of Stavanger, and the Norwegian Centre for Movement Disorders, Stavanger University Hospital, Stavanger, Norway; 2 Research Professor, Nordic Institute for Chiropractic and Clinical Biomechanics, part of Clinical Locomotion Science, University of Southern Denmark, Odense, Denmark; 3 Private Practice, Kangasala, Finland; 4 Private Practice, Helsinki, Finland; 5 Private Practice, Tampere, Finland; 6 Private Practice, Lahti, Finland; 7 Consultant, The Back Research Centre, part of Clinical Locomotion Science, University of Southern Denmark, Ringe, Denmark

## Abstract

**Background:**

In a previous Swedish study it was shown that it is possible to predict which chiropractic patients with persistent LBP will not report definite improvement early in the course of treatment, namely those with LBP for altogether at least 30 days in the past year, who had leg pain, and who did not report definite general improvement by the second treatment. The objectives of this study were to investigate if the predictive value of this set of variables could be reproduced among chiropractic patients in Finland, and if the model could be improved by adding some new potential predictor variables.

**Methods:**

The study was a multi-centre prospective outcome study with internal control groups, carried out in private chiropractic practices in Finland. Chiropractors collected data at the 1st, 2^nd ^and 4^th ^visits using standardized questionnaires on new patients with LBP and/or radiating leg pain. Status at base-line was identified in relation to pain and disability, at the 2^nd ^visit in relation to disability, and "definitely better" at the 4^th ^visit in relation to a global assessment. The Swedish questionnaire was used including three new questions on general health, pain in other parts of the spine, and body mass index.

**Results:**

The Swedish model was reproduced in this study sample. An alternative model including leg pain (yes/no), improvement at 2^nd ^visit (yes/no) and BMI (underweight/normal/overweight or obese) was also identified with similar predictive values. Common throughout the testing of various models was that improvement at the 2^nd ^visit had an odds ratio of approximately 5. Additional analyses revealed a dose-response in that 84% of those patients who fulfilled none of these (bad) criteria were classified as "definitely better" at the 4^th ^visit, vs. 75%, 60% and 34% of those who fulfilled 1, 2 or all 3 of the criteria, respectively.

**Conclusion:**

When treating patients with LBP, at the first visits, the treatment strategy should be different for overweight/obese patients with leg pain as it should be for all patients who fail to improve by the 2^nd ^visit. The number of predictors is also important.

## Background

The causes of non-specific low-back pain (LBP) are largely unknown [1,2]. Obviously, this is a hindrance to a rational approach to both prevention and treatment. In general, both etiologic studies and randomized controlled clinical trials are based on the concept that non-specific LBP is one single entity. However, most clinicians with an interest in back pain probably consider it to consist of several specific conditions, which have not been properly recognized, understood and described.

Chiropractors in the Nordic countries use predominantly spinal manipulative therapy (SMT) in their treatment of back problems, frequently in combination with soft tissue therapy, advice on exercise, ergonomic precautions, and lifestyle changes [3-5]. Randomized controlled clinical trials have shown that SMT has a positive effect on LBP [6]. However, overall, the magnitude of the effect seems to be relatively small. Those, who believe that back pain consists of several specific but (as yet) undefined subgroups, obviously think that the recognition of these would improve the quality of care and that the selection of homogeneous study populations in etiological studies and clinical trials would improve the quality of research.

Until recently it has not been documented which patients with LBP are most likely to benefit from the chiropractic approach. However, the predictive value of a set of clinical observations has been previously studied in patients with LBP receiving chiropractic care [7-10]. This research, conducted in Norway and Sweden under the Nordic Back Pain Subpopulation Program, has been running over the past years, in which specific subgroups of patients with LBP are systematically studied. For instance, it was shown that it is possible to predict which chiropractic patients with persistent LBP will not report definite improvement early in the course of treatment, making it possible to exclude from treatment those who are unlikely to become LBP-free.

Furthermore, early recovery at the 4^th ^visit was noted to be a predictor for outcome 3 and 12 months later [7] and the status already by the second visit predicted status at the fourth visit [10].

Specifically, in a Swedish study of patients with LBP, it was shown that patients with LBP for altogether at least 30 days in the past year, who had leg pain, and who did not report some improvement by the second treatment, were not good candidates for definite improvement by the 4^th ^visit [10]. Although the final model was excellent in predicting non-response at the 4^th ^visit (96%), it could only predict 19% of patients who would be "definitely better".

The objectives of the present study were to investigate if similar findings could be reproduced in a different cultural setting (Finland), and if the model could be improved by adding a few more potential predictors.

## Methods

### Design

The study was designed as a multi-centre clinic-based prospective outcome study with internal control groups, using standardised questionnaires, conducted in private chiropractic practices in Finland.

### Planning the study

A steering group was established, consisting of five researchers and one research officer, supervised by an experienced researcher. Questionnaires from the previous Swedish study were used by permission, translated and culturally adapted in a pilot-study involving 30 patients for face validity.

Based on clinical intuition, three variables were added to this questionnaire. These were weight/height (body mass index-BMI), general health, and pain in other parts of the spine.

### Study participants – chiropractors

All members of the Finnish Chiropractic Union were invited to participate in the study to collect data from a maximum of 40 patients each. The steering group members instructed and assisted the involved chiropractors using a method previously described by a Swedish research group [10], with one person in the team (SM) being responsible for the logistics of the study.

### Study participants – patients

Consenting patients were included after receiving information on the purpose of the study by their chiropractor. Inclusion criteria were new patients with LBP with or without leg pain and patients had to return at least once following the first visit.

### Ethics

Clinician and patient anonymity was ensured by using codes, tying the patient to the treating chiropractor. This code was destroyed after the 4^th ^treatment visit. Only the treating chiropractor knew the identity of the participating patients. The regional scientific ethics committee reviewed and defined this study as a quality assurance project, which does not require committee approval.

### Data collection

Information for the study was collected by the chiropractors on the first, second, and fourth visits [Additional files 1, 2, 3]. For patients whose treatments were completed before the fourth visit, the last information was provided at the time of the final treatment. The whole collection period took place between the months of March and August 2005. Intervention was chiropractic management as decided by the treating chiropractor.

### Variables of interest

All potential predictors but three were taken from the previous Swedish study [10], consisting of the base-line variables plus information obtained at the return visit in relation to whether there was at least one reported item of improvement as compared to at base-line in relation to pain when turning in bed, sleeping, putting on socks/shoes, walking, or getting up from sitting. This new variable was named *better at 2^nd ^visit*. Another new variable (*number of disabilities*) was created by counting the number of positive answers to these questions (pain when turning in bed, etc.). Three new items: *BMI*, *general health*, and *pain in other parts of the spine*, were also included in the questionnaire.

Information on time since last treatment, both at the 2^nd ^and 4^th ^visit, and type of treatment provided at the first visit was also collected to describe the patients and the clinical procedure. Also these questions were taken from the previous Swedish study [10]. *Severity of pain *was reported at all three times to enable comparisons over time, using a five point scale ranging from unbearable to pain free. Another of the descriptive variables was *unsuitable reactions*. A local pain reaction after the first treatment was defined as "unsuitable" if it was reported to have lasted for longer than 24 hrs, or if it consisted of new radiating pain (regardless duration), according to standardized answers, based on information from two previous descriptive studies of Norwegian and Swedish patients who received chiropractic treatment [11,12].

In addition, reactions described as free text under "other" were individually scrutinized for unsuitable reactions.

The outcome (global assessment of present status at the 4^th ^visit) was defined as positive only for those patients who reported to be *definitely better *at the fourth visit (or at the last visit if treatment was ended before the fourth visit). Missing data for this variable were interpreted as not being definitely better, i.e. a form of worst case interpretation was used.

### Validation procedures

The pilot study showed good compliance and understanding of the questionnaires by the patients, indicating good face validity. The outcome variable was validated against the pain reporting at the 4^th ^visit and found to be satisfactory [Table 1]. Thus we noted that 95% of those who reported to be definitely better also said that they had no pain (61%) or mild pain (34%).

**Table 1:** Cross-tabulation of the variables "General Improvement" and "Present Pain Status" at the 4^th ^visit. Percentages in brackets.

GENERAL IMPROVEMENT	No pain	Mild pain	Moderate pain	Severe pain	Unknown	Total
Definitely better	395 (61)	222 (34)	27 (4)	2 (< 1)	6 (1)	652 (100)

Probably better	17 (12)	77 (57)	39 (29)	2 (1)	1 (1)	136 (100)

Unchanged	0 (0)	8 (16)	28 (55)	15 (29)	0 (0)	51 (100)

Probably worse	0 (0)	0 (0)	5 (56)	3 (33)	1 (11)	9 (100)

Definitely worse	0 (0)	0 (0)	0 (0)	0 (0)	1 (100)	1 (100)

Unknown	2 (1)	1 (1)	0 (0)	0 (0)	132 (98)	135 (100)

Total	414 (42)	308 (31)	99 (10)	22 (2)	141 (14)	984 (100)

Data were cleaned and investigated for data entry errors. A random selection of 100 questionnaires was checked manually, in which no data entry errors were found. However, later it was discovered that in a small number of patients weight and height data had been switched by the informants. These incorrect values were easily detected and corrected.

### Data management and analysis

Each variable was described and where relevant collapsed into a smaller number of categories. *Height *and *weight *were transformed into *BMI*, which was classified into underweight, normal weight, over weight and obesity, taking into account the age of the subjects [13]. *BMI *and *age *were transformed into categorical variables. Thereafter, bivariate analyses were carried out of all independent variables vs. the outcome variable. Associations were considered to be statistically significant if *p *was equal to or smaller than 0.05 and these were later used in the multivariate analyses.

Two sets of multivariate analyses were carried out (logistic regression). In the first, we used the same variables as those found to be significant in the previous Swedish study, to see if their results could be reproduced in the present study sample. These variables were *leg pain, duration of pain in the past year *and *improvement at the 2^nd ^visit*. In the second analysis, all the potential predictors used in the present study, shown to be significantly associated with the outcome variable, were entered into a logistic regression. Non-significant variables were removed until only significant variables remained. Because of the relatively large study sample, the significance level was set at *p *= 0.05 for allowing the variable to enter the model. In the second analysis, the three additional variables were also taken into account *BMI*, *pain in other parts of the spine*, and *general health*.

For each model, odds ratios with their 95% confidence intervals were calculated as well as the sensitivity, specificity, numbers correctly classified, and area under the Receiver Operator Characteristic curve. A Receiver Operator Characteristic value of 50% indicates chance findings, whereas a minimal value of at least 70%, arbitrarily, is considered to be acceptable, and a value of 100% indicates perfection. In all analyses, adjustment was made for clustering, to counteract the undue effect single clinicians could have on the results.

## Results

### Response rate

At baseline, all 47 eligible chiropractors in the Finnish Chiropractic Union were invited to participate in the study to include 40 patients each. The maximum possible amount of patients was 1880. Thirty-three chiropractors participated, which means that the optimal amount of patients was 1320. These chiropractors returned complete sets of questionnaires from 1023 patients. From the 1023 returned questionnaires, 13 were discarded due to incorrect coding and a further 22 were discarded due to missing relevant baseline data and 4 because they appeared to belong to patients who had neither LBP nor leg pain. Occasionally, some data were missing for the various variables.

### At base-line

The base-line sample has been described in Table 2, and the main findings are described below. Of the final 984 participants (74.5% of the optimal study sample), there were 506 men and 471 women, whereas information was missing for the remaining 7 persons. The age ranged from 8 to 90 and the largest age-groups were 21 to 50 years (60%). The mean and median age was 45.5 and 44 years, respectively.

**Table 2:** Base-line description of 984 patients.

Variable	Subgroups	Frequency	Percentage
Sex	Men	506	52
	Women	471	48
	Not stated	7	< 1

Age	0–20	43	4
	21–50	586	60
	≥ 50	355	36

LBP	Yes	961	98
	No	23	2

Leg pain	Yes	461	47
	No	523	53

Pain intensity at baseline	None	16	2
	Mild	183	19
	Moderate	443	45
	Severe	281	29
	Unbearable	59	6

Days with pain at baseline	Max 2 wks	363	37
	> 2 wks	621	63

Constant pain past year	Yes	637	65
	No	347	35

Days with pain past yr	< 30 days	437	44
	≥ 30 days	547	56

Pain turning in bed	Yes	515	53
	No	452	47

Pain when sleeping	Yes	397	41
	No	570	59

Pain putting on socks/shoes	Yes	639	66
	No	334	34

Pain in walking	Yes	527	54
	No	443	46

Pain getting up from sitting	Yes	686	70
	No	290	30

Number of disabilities	0	76	8
	1	141	15
	2	191	20
	3	192	20
	4	178	19
	5	174	18

General health	Excellent/good	924	94
	Less than good	54	6

Pain in neck and/or mid-back past year	No	444	45
	Yes < 30 days	281	29
	Yes ≥ 30 days	247	25

Body mass index	Underweight/Normal weight	455	47
	Overweigh/obese	512	53

Better directly after treatment	Yes	635	67
	No	317	33

**Figure 1:**
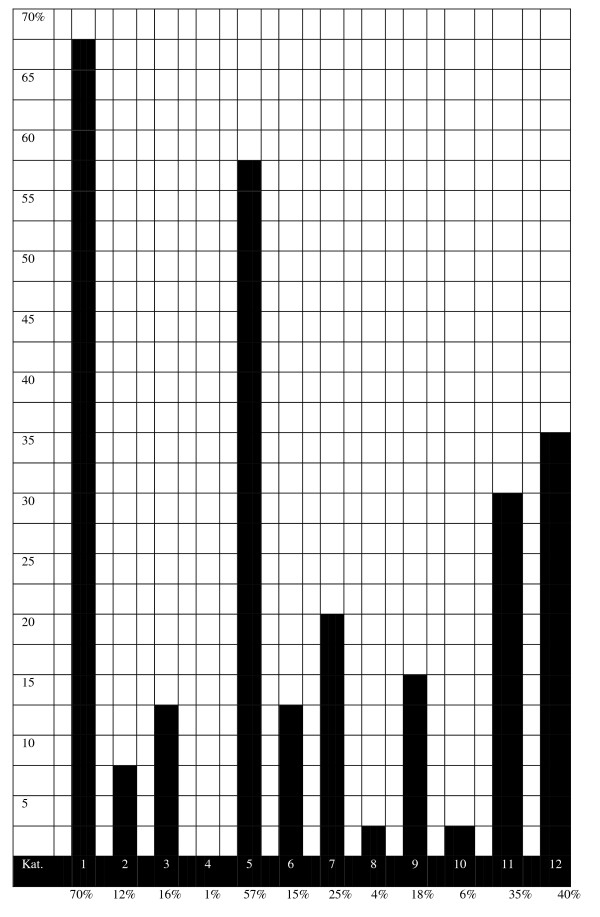
**The prevalence of 12 different subgroups of LBP in Finnish chiropractic patients**. The subgroups are ordered from the most benign to the more severe to add up to 100% (n = 977). Groups: 1 – baseline 1 week, non-persistent, intermittent; 2 – baseline 1 week, non-persistent, daily; 3 – baseline 1 week, persistent, intermittent; 4 – baseline 1 week, persistent, daily; 5 – baseline 2 weeks, non-persistent, intermittent; 6 – baseline 2 weeks, non-persistent, daily; 7 – baseline 2 weeks, persistent, intermittent; 8 – baseline 2 weeks, persistent, daily; 9 – baseline > 2 weeks, non-persistent, intermittent; 10 – baseline > 2 weeks, non-persistent, daily; 11 – baseline > 2 weeks, persistent, intermittent; 12 – baseline > 2 weeks, persistent, daily. • "base-line" refers to the duration of pain at the first visit. • "non-persistent" = altogether < 30 days in the past year. • "persistent" = altogether at least 30 days in the past year. • "intermittent" and "daily" refers to the pain pattern at the first visit.

At base-line, 98% had LBP and almost half had leg pain. Pain was most commonly reported as moderate (45%) or severe (29%), and 63% had experienced pain for at least 2 weeks. At the time of consultation, the nature of the pain was described as constant by 65% and a little more than half had experienced the pain for altogether at least more than 30 days in the past year. The spread of data is shown for the various combinations of the three variables *duration of pain at base-line, constant/not constant pain at base-line*, and *duration of pain in the past year *[Figure 1].

Sixty-nine percent reported between 2 and 4 painful *number of disabilities *out of 5 possible, with pain getting up from *sitting *being most common (70%), followed by pain putting on *socks/shoes *(66%), and pain on *walking *(54%).

Almost all reported to have excellent or good general health, and 25% reported altogether at least 30 days of pain in the neck or mid back in the past year. The group was almost equally distributed between underweight/normal weight and overweight/obese. Two-thirds reported to feel immediately better after the 1st treatment.

### At the return visit

As can be seen in Table 3, 70% returned for their second visit within 1 week. Almost all had received SMT at the first visit, and 61% received soft tissue therapy. A drop table was used in 44% and pelvic block in 25% of patients, whereas the sacro-occipital technique was virtually non-existing (1%).

**Table 3:** Follow-up data at the 2^nd ^visit

Variable	Subgroups	Frequency	Percentage
Number of days since 1^st ^treatment	1 d	43	4
	2–6 d	518	53
	7 d	172	17
	1–2 wks	96	10
	> 2 wks	77	9
	Not stated	78	8

SMT at 1^st ^visit	Yes	898	91
	No	53	5
	Not stated	33	3

Drop table at 1^st ^visit	Yes	433	44
	No	517	52
	Not stated	34	3

Soft tissue therapy at 1^st ^visit	Yes	600	61
	No	350	36
	Not stated	34	3

Pelvic blocks at 1^st ^visit	Yes	248	25
	No	702	71
	Not stated	34	3

Sacro-Occipital technique at 1^st ^visit	Yes	6	1
	No	944	96
	Not stated	34	3

Other technique at 1st visit	Yes	191	19
	No	759	77
	Not stated	34	3

Intensity of pain at 2^nd ^visit	No pain	155	16
	Mild	440	45
	Moderate	271	28
	Severe	49	5
	Unbearable	8	1
	Not stated	61	6

At least one unsuitable reaction	No	832	85
	Yes	152	15

Definitely better in at least one disability aspect (turn in bed, put on socks/shoes etc.)	Yes	558	57
	No	426	43

The most commonly reported intensity of pain was now mild (45%) or moderate (28%) and 85% reported to have experienced no "unsuitable reaction". Fifty-seven percent reported to have improved in at least one "disability" aspect (turn in bed, put on socks/shoes etc.).

### At the fourth visit

The most commonly reported duration since the first visit was maximum 2 weeks (42%). The intensity of pain was now even more reduced, most commonly reported as none (42%) or mild (31%).

Two-thirds reported to be definitely better, 20% reported to be less than definitely better whereas the outcome was unknown for 14%. The latter group was classified as not definitely better [Table 4].

**Table 4:** Data from the fourth visit

Variable	Subgroups	Frequency	Percentage
Number of days since first treatment	Max. 2 wks	413	42
	2–4 wks	284	29
	4–6 wks	67	7
	6–8 wks	46	5
	More	33	3
	Unknown	141	14

Intensity of pain at 4^th ^visit	No pain	414	42
	Mild	308	31
	Moderate	99	10
	Severe	22	2
	Unbearable	0	0
	Unknown	141	14

Global assessment of present status	Definitely better	652	66
	Not definitely better (i.e. probably better, unchanged, probably worse, definitely worse)	197	20
	Unknown	135	14

### Bivariate analyses – the independent variables vs. the outcome variable

The following variables were positively associated with definite improvement at the 4^th ^visit: *Leg pain*, *duration of pain at base-line, total duration of pain in the past year, general health, other spinal pain in the past year, BMI, immediate improvement *and *better at the 2^nd ^visit*.

Consequently, there were no significant associations for the following variables: *Sex, age, severity of pain at base-line, constant pain at base-line, pain turning in bed, problems sleeping, problems putting on socks/shoes, pain on walking, pain on getting up from sitting*, and *number of "disabilities"*.

### Multivariate analyses – testing the Swedish model

As can be seen in Table 5, the original "best" Swedish model, consisting of the three variables *leg pain*, *duration of pain in the past year*, and *better at the 2^nd ^visit*, when tested on our data obtained a sensitivity of 41%, a specificity of 87%, and numbers correctly classified were 71.5%. The area under the Receiver Operator Characteristic curve was 72%.

**Table 5:** Multivariate analyses testing associations with the outcome variable. Significant findings are in bold.

Models	Variables tested	OR and 95% CI	• Sensitivity • Specificity • Numbers correctly classified • Area under the ROC
"Best" Swedish model re-tested, according to previous study	**Leg pain**	**1.6 (1.2–2.1)**	41%, 87%, 71.5%, 72%

	Duration of pain past yr	1.1 (0.8–1.6)	

	**Better at 2^nd ^visit**	**4.7 (3.4–6.6)**	

"Full" Swedish model, i.e. including significant variables that had been included in previous study	**Leg pain**	**1.5 (1.1–2.0)**	47%, 83%, 71%, 72%

	Duration of pain past yr	1.2 (0.8–1.7)	

	**Better at 2^nd ^visit**	**4.7 (3.4–6.6)**	

	Duration of pain at base-line	1.0 (0.7–1.3)	

	**Immediate improvement**	**1.3 (1.0–1.7**)	

Improved Swedish model, i.e. removing irrelevant variables from the model above	**Leg pain**	**1.6 (1.2–2.2**)	41%, 87%, 72%, 71%

	**Better at 2^nd ^visit**	**4.8 (3.5–6.8)**	

Final minimal Swedish model, i.e. retaining the "best" variable	**Better at 2^nd ^visit**	**4.9 (3.6–6.8**)	68%, 69%, 69%, 69%

Full Finnish model, i.e. allowing for the three new variables included in the present study	**Leg pain**	**1.4 (1.0–2.0)**	52%, 83%, 73%, 73%

	Duration of pain past yr	1.1 (0.8–1.6)	

	**Better at 2^nd ^visit**	**5.0 (3.5–7.1**)	

	Duration of pain at base-line	0.9 (0.7–1.3)	

	Immediate improvement	1.2 (0.9–1.6)	

The full Swedish model including the five variables, which in the present study were significantly associated with the outcome, did not result in better values.

**Figure 2:**
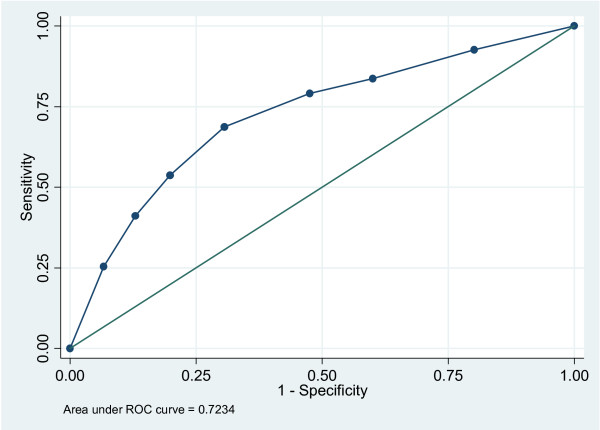
**The Receiver Operator Characteristic curve**. The final minimal model, based on the variables previously used in the Swedish study, consisted of only one variable, *better at 2*^*nd *^visit.

The final minimal model, based on the variables previously used in the Swedish study, consisted of only one variable, *better at 2*^*nd *^visit. It had a somewhat higher sensitivity and lower specificity but there was almost no change in the number classified and area under Receiver Operator Characteristic curve [Figure 2].

### Multivariate analyses – adding the three new variables

The three new variables, *BMI, general health*, and *spinal pain*, were added to the full model as described above [Table 6]. Again the estimates of clinical significance changed somewhat, but the presence of these extra three factors did not really improve the model. *BMI *was retained in the final model together with *leg pain *and *better at 2^nd ^visit*.

**Table 6:** Multivariate analyses testing associations with the outcome variable. Significant findings written in bold.

Models	Variables tested	OR and 95% CI	• Sensitivity • Specificity • Numbers correctly classified • Area under the ROC
	General health	1.1 (0.6–2.1)	

	**BMI**	**1.4 (1.0–2.0)**	

	Other spinal pain past yr - yes < 30 d - yes > 30 d	0.7 (0.4–1.1) 1.0 (0.7–1.4)	

Final minimal Finnish model, i.e. retaining the "best" variables	**Leg pain**	**1.6 (1.2–2.1)**	54%, 80%, 71%, 72%

	**Better at 2^nd ^visit**	**5.0 (3.6–7.1)**	

	**BMI**	**1.4 (1.0–2.0)**	

### Multivariate analyses – from a clinical perspective

In all models, *better at the 2^nd ^visit *in relation to outcome had the strongest odds ratio with estimates between 4.7 and 5.0. For detailed information, see Table 6. In the clinical situation, this means that 80% of patients with LBP with or without radiating leg pain, who report to be better at the second visit, are definitely improved by the 4^th ^visit, whereas this is the case only for 50% of those who are not better by the second visit.

### Post hoc analyses

Three additional exploratory analyses were undertaken. First, in order to see if the *type of treatment *at the first visit (SMT, STT, drop-piece, blocks, SOT, and other) would have an observable effect on the outcome variable, or improvement at the 2^nd ^visit, but no such findings emerged (data not shown).

Second, an attempt was made to see if *duration since the 1st visit *(at the 4^th ^visit) was of any relevance for the outcome. This variable was therefore categorized into 1–14 days, 14–28 days, and one month or more and forced into the final Finnish model. However, it was not significantly associated with outcome and its presence did not significantly alter the estimates in the model (data not shown).

Finally, a logistic regression was undertaken in which the 3 variables that remained in the final model (*leg pain, not better at 2^nd ^visit*, and *overweight/obese*) were checked for a dose-response, in relation to being definitely improved at the 4^th ^visit. With none of these findings, 84% would be definitely better at the 4^th ^visit, whereas the corresponding figures for one, two, respectively three of these findings were 75%, 60% and 34%. The data have been presented also as odds ratios in Table 7.

**Table 7:** Predictor variables were tested against outcome at the 4^th ^visit. The predictor variables were: overweight or obese, leg pain, and not better at 2^nd ^visit.

Number of predictor variables present in the patient	OR (95% CI) in relation to not being definitely improved at the 4th visit*
0 (index)	1

1	1.8 (1.1–2.9)

2	3.4 (2.1–5.6)

3	10.2 (5.8–18.1)

* The Hosmer-Lemeshow test for goodness-of-fit revealed that this model had a perfect (100%) fit.

## Discussion

The results of the present study confirm that it is possible to predict short-term outcome in patients with LBP who receive chiropractic care. This is a clinically relevant finding, as it has been previously shown that short-term outcome (i.e. recovery by the fourth visit) is a predictor for the outcome at both 3 and 12 months, at least in patients with relatively long-lasting or recurrent LBP [7].

When the previously achieved best Swedish model was applied to patients from Finland, the associations between outcome and the three relevant variables (leg pain, duration of pain in the past year and leg pain) were again positive, although duration failed to reach significance and leg pain was only weakly associated, and in the final analysis, only improvement at the second visit remained significant with an odds ratio of 4.9.

Improvement at the second visit meant that patients reported that at least one of the five "disabilities" was better than at base-line, namely sleeping, turning in bed, putting on socks/shoes, getting up from a chair, or walking.

Even when adding the three new factors (BMI, other spinal pain and general health), improvement at the second visit was the only strongly associated variable that emerged from the multivariate analysis, still with an odds ratio of 5.

In the final analysis, taking into account also leg pain and BMI did not really improve the estimates in a clinically meaningful way. However, when the number of these predictor variables present in each person was tested against outcome, a dose-response was revealed. In the whole study sample, the proportion of patients in the study who were "definitely better" at the fourth visit was 66%. In patients with none of these three predictors, 84% were better, whereas only 34% of those who had all three belonged to this category.

Obviously, it is important to keep in mind the weaknesses in this type of study design, such as several possibilities for bias in relation to selection of practitioners and patients, in relation to their expectations of treatment outcome, and in relation to the recording of outcome, such as there being a tendency to "inflate" the result by the chiropractor in questionnaire studies like this one and patients providing polite positive answers. To counteract the latter possibility, patients were not considered improved unless they had stated that they were "definitely" improved. Also, clinicians were informed that the purpose of the study was to study differences between patients who react differently to the treatment, to counteract any desire to "prove" a high success rate. It was also impossible to define the exact nature of "leg pain" due to the brief questionnaire.

Clinical studies frequently investigate outcome by a large number of research tools, such as visual analogue scales indicating level of pain and disability questionnaires. Also, it is considered important that outcome data are collected by people who are independent to the treatment procedure, or at least using self-report questionnaires. However, when considering the feasibility of this type of study, one has to balance the negative aspects with the present approach (i.e. the risk of reporting bias and the inconvenience of brief outcome measures) against its positive aspects (high participation and clinically relevant outcome measures). In our study group, we are depending on clinicians to participate in their normal clinical context, without financial compensation for time lost due to lengthy procedures, which obviously requires the use of a very short questionnaire. Also, most private practitioners probably use and relate well to our outcome measure "definitely better", which makes the results of our study more easily applicable in clinical practice.

The reader should also be aware of the fact that with no control group, these outcome data cannot be regarded as estimates of treatment effect. The purpose of the study is instead to study the effect that various factors seem to have on the outcome, bearing in mind that the predictors tested in this study possibly could give similar results in patients who are treated with other therapies or perhaps even in those who receive no treatment at all. Obviously this would have to be tested in randomised controlled clinical trials. Interesting future research areas would also be to study the effect of various management strategies (e.g. frequent vs. less frequent treatments) and to investigate also the effect on outcome of different various psychological profiles.

Strengths in this study are the large study sample, and the good quality of the data. There were only few obviously faulty questionnaires and only few missing data. Positive aspects of this type of study are that it documents the normal clinical situation and that it includes a wide variety of practitioners and patients. Secondary gains are that it makes chiropractors able to participate in research without having to spend too much time with the project, makes them aware of the rigours associated with data collection, encourages an interest in the study results, and hopefully, makes research results more clinically relevant for those who participated in data collection. Although this study design requires a simplistic approach to data collection, it is a relatively cheap way to collect clinically relevant information on a large number of patients.

## Conclusion

There are three important messages in this report. First, already at the first visit one should be vigilant with overweight/obese patients who have pain radiating into the leg. Second, at the return visit, for these patients if there is lack of improvement, the short-term prognosis is poor. Third, that any patient, who fails to improve at the 2^nd ^visit has a poor short-term prognosis. Therefore, when treating patients with LBP, the treatment strategy should be different for overweight/obese patients with leg pain as it should be for all patients who fail to improve by the 2^nd ^and 4^th ^visits.

## Competing interests

The authors declare that they have no competing interests.

## Authors' contributions

SM was responsible for planning and executing the study, participated in the data collection and drafted the manuscript. CLY supervised the process. Both SM and CLY participated in the design of the study and performed the analysis together with NW. NW was responsible for the statistical analysis. TA, OA, KE, MT and HP participated in the design of the study and the data collection. All authors read, finalized and approved the final manuscript.

## Supplementary Material

Additional file 1 Baseline questionnaire for Finnish predictor study (pdf). Finnish distributed questionnaire followed by an English translation. Click here for file

Additional file 2 Return questionnaire for Finish predictor study (pdf). Finnish distributed questionnaire followed by an English translation. Click here for file

Additional file 3 4^th ^visit questionnaire for Finnish predictor study (pdf). Finnish distributed questionnaire followed by an English translation. Click here for file
