# Supplementary material for: The Nordic back pain subpopulation program: predicting outcome among chiropractic patients in Finland
Source: Chiropr Osteopat. 2008 Nov 7;16:13. doi: 10.1186/1746-1340-16-13 (PMC2588613; doi:10.1186/1746-1340-16-13)
Supplement: Additional file 1 — Baseline questionnaire for Finnish predictor study (pdf). Finnish distributed questionnaire followed by an English translation. [file 1746-1340-16-13-S1.pdf]

**PERUSTIEDOT** **Potilas No.:__________**

Rastita / täytä sopivin vaihtoehto.

**1. Sukupuoli** ☐Mies ☐Nainen

**2. Ikä ______vuotta**

**3. Oire**

a) Kipu alaselässä ☐ Kyllä ☐ Ei
b) Kipu jalassa ☐ Kyllä ☐ Ei

**4. Kivun voimakkuus edellisen vuorokauden aikana**

☐ Ei kipua ☐ Lievä ☐ Kohtalainen ☐ Kova ☐ Sietämätön

**5. Tämän hetkisten oireiden kesto**

☐ 1 – 7 vrk ☐ 8 – 14 vrk ☐ Yli 14 vrk

**6. Oireiden kokonaiskesto kaikkiaan edellisen vuoden aikana**

☐ Korkeintaan 30 päivää, ajoittainen
☐ Korkeintaan 30 päivää, päivittäin
☐ Yli 30 päivää, ajoittainen
☐ Yli 30 päivää, päivittäin

**7. Onko potilaalla vaikeuksia**

a) kääntyä sängyssä? ☐ Kyllä ☐ Ei
b) nukkua? ☐ Kyllä ☐ Ei
c) laittaa sukkia/kenkiä? ☐ Kyllä ☐ Ei
d) kävellä? ☐ Kyllä ☐ Ei
e) nousta istumasta? ☐ Kyllä ☐ Ei

**8. Havaitsiko potilas parannusta olossaan välittömästi hoidon jälkeen?**

☐ Kyllä ☐ Ei ☐ Ei hoidettu ensikäynnillä

**9. Millainen on potilaan terveydentila yleisesti ottaen?**

☐ Erinomainen
☐ Aika hyvä
☐ Aika huono
☐ Erittäin huono
☐ En osaa sanoa

**10. Onko potilas viimeisen vuoden aikana kärsinyt alaselkäkivun lisäksi muista selkä- tai niskavaivoista**

☐ En
☐ Kyllä, yhteensä 30 päivää tai vähemmän
☐ Kyllä, yhteensä yli 30 päivää

**11. Potilaan paino: ______kg**

**12. Potilaan pituus: ______cm**

---

(English translation)

**Base-Line interview:**

**1. Sex:** Male/ female

**2. Age:** …..years

**3. Symptom:** Low back pain Yes/No
Leg pain Yes/No

**4. Intensity of pain during the past 24 hours?**

No pain/Mild/Moderate/Severe/unbearable

**5. Duration of present symptoms?**

1-7 days
8-14 days
More than 14 days

**6. The overall duration of symptoms during the past year:**

No more than 30 days, not constant
No more than 30 days, daily
More than 30 days, not constant
More than 30 days, daily

**7. Has the patient difficulties in…**

| | |
|---|---|
| …turning in bed | Yes/No |
| …sleeping | Yes/No |
| …putting the socks/shoes on | Yes/No |
| …walking | Yes/No |
| …standing up from sitting | Yes/No |

**8. Did the patient feel better right after treatment?**

Yes/No/No treatment at first visit

**9. Patients weight** ……. kg

**10. Patients height** ……. cm
