# Supplementary material for: The Nordic back pain subpopulation program: predicting outcome among chiropractic patients in Finland
Source: Chiropr Osteopat. 2008 Nov 7;16:13. doi: 10.1186/1746-1340-16-13 (PMC2588613; doi:10.1186/1746-1340-16-13)
Supplement: Additional file 2 — Return questionnaire for Finish predictor study (pdf). Finnish distributed questionnaire followed by an English translation. [file 1746-1340-16-13-S2.pdf]

Suunniteltu haastattelu tietojen saamiseksi seuraavalla käynnillä (tai ensimmäisen hoidon jälkeen) **Potilas No.:_________**

Rastita / täytä sopivin vaihtoehto

1. Kuinka kauan on ensimmäisestä hoidosta?

☐ 1 päivää	☐ 2 päivää	☐ 3 päivää
☐ 4 päivää	☐ 5 päivää	☐ 6 päivää
☐ 7 päivää	☐ 8-14 päivää	☐ > 14 päivää

2. Alaselän/lantion hoitomuoto edellisellä hoidolla (useat vaihtoehdot mahdollisia)?

☐ Selkärangan manipulaatiohoito (mukaan lukien Aktivaattori)
☐ Drop
☐ Pehmytkudoshoidot
☐ Kiilat
☐ SOT

☐Muu, mikä?____

3. Kuinka voimakasta kipu on ollut edellisen vuorokauden aikana?

☐ Ei kipua
☐ Lievä
☐ Kohtalainen
☐ Kova
☐ Sietämätön

4. Oliko potilaalla epämiellyttäviä tuntemuksia hoidon jälkeen?

**A.** Paikallinen kipu	☐ Kyllä	☐ Ei
Jos kyllä, kauanko se kesti?	☐ Korkeintaan vrk	☐ >vrk
**B.** Uusi säteilykipu	☐ Kyllä	☐ Ei
Jos kyllä, kuinka voimakas?	☐ Lievä<br>☐ Kova	☐ Kohtalainen<br>☐ Sietämätön
**C.** Väsymystä	☐ Kyllä	☐ Ei
Jos oli, kuinka kauan se kesti?	☐ Korkeintaan vrk	☐ >vrk

D. Muuta ____

5. Jos potilaalla oli oireita seuraavissa toiminnoissa, ovatko nämä vähentyneet kivun lieventymisen vuoksi viimeisen vuorokauden aikana?

A. ☐ Sängyssä kääntyminen

Selvästi☐ Todennäköisesti☐ Muuttumaton☐ Huonompi☐ Asiaankuulumaton☐

B. ☐ Nukkuminen

Selvästi☐ Todennäköisesti☐ Muuttumaton☐ Huonompi☐ Asiaankuulumaton☐

C. ☐ Kenkien/sukkien pukeminen

Selvästi☐ Todennäköisesti☐ Muuttumaton☐ Huonompi☐ Asiaankuulumaton☐

D. ☐ Kävely

Selvästi☐ Todennäköisesti☐ Muuttumaton☐ Huonompi☐ Asiaankuulumaton☐

E. ☐ Istumasta nouseminen

Selvästi☐ Todennäköisesti☐ Muuttumaton☐ Huonompi☐ Asiaankuulumaton☐

F. ☐ Yleisolotila

Selvästi☐ Todennäköisesti☐ Muuttumaton☐ Huonompi☐

(English translation)

Planned interview for the return visit

1. Time elapsed since the first treatment?

1day/2days/3days/4days/5days/6days/7days/8-14days/more than 14 days

2. Low back/pelvic treatment method at previous treatment session (you can choose more than one)?

Spinal manipulative therapy (including activator)/Drop/Soft tissue work/Blocking or SOT/Other… which?

3. The intensity of pain during the past 24 hours?

No pain/Mild/Moderate/Severe/Unbearable

4. Did the patient have any unpleasant reactions after the treatment? E.g.:

1. Local pain	Yes/No
If yes, how long did it last?	No more than ….. days
2. New radiating pain?	Yes/No
If yes, how severe	Mild/Moderate/Severe/Unbearable
3. Tiredness	Yes/No
If yes, how long did it last?	No more than ….. days.
4. Something else?	………………………..

5. If the patient experienced symptoms in the following activities/functions, have these symptoms diminished because of the decreased pain during the past 24 hours?

Turning in bed

Clearly relieved/Maybe relieved/Unchanged/Worse/Meaningless (not applicable)

Sleeping

Clearly relieved/Maybe relieved/Unchanged/Worse/ Meaningless

Putting on shoes/socks

Clearly relieved/Maybe relieved/Unchanged/Worse/ Meaningless

Walking

Clearly relieved/Maybe relieved/Unchanged/Worse/ Meaningless

Standing up from sitting

Clearly relieved/Maybe relieved/Unchanged/Worse/ Meaningless

General feeling

Clearly relieved/Maybe relieved/Unchanged/Worse/ Meaningless
