# Supplementary material for: The Nordic back pain subpopulation program: predicting outcome among chiropractic patients in Finland
Source: Chiropr Osteopat. 2008 Nov 7;16:13. doi: 10.1186/1746-1340-16-13 (PMC2588613; doi:10.1186/1746-1340-16-13)
Supplement: Additional file 3 — 4th visit questionnaire for Finnish predictor study (pdf). Finnish distributed questionnaire followed by an English translation. [file 1746-1340-16-13-S3.pdf]

Suunniteltu haastattelu tietojen saamiseksi neljännellä (tai aiemmin jos hoito loppuu ennemmin) **Potilas No.: ________**

1. Kuinka kauan on ensimmäisestä hoidosta?

☐ 1 - 14 päivää
☐ 15 – 28 päivää
☐ 4 - 6 viikkoa
☐ 6 – 8 viikkoa
☐ Yli 8 viikkoa

2. Potilaan kivun voimakkuus edellisen vuorokauden aikana?

☐ Ei kipua
☐ Lievä
☐ Kohtalainen
☐ Kova
☐ Sietämätön

3. Tilanne (verrattuna ensimmäiseen käyntiin)?

☐ Selvästi parempi
☐ Todennäköisesti parempi
☐ Muuttumaton
☐ Todennäköisesti huonompi
☐ Varmasti huonompi

4. Jos potilasta ei hoidettu neljää kertaa, niin kuinka monta kertaa potilasta hoidettiin?

☐ 1 ☐ 2 ☐ 3

5. Miksi hoito lopetettiin ennen neljättä käyntiä?

☐ Tarpeeton – OK
☐ Ei palannut

☐ Ohjattu muualle.

Miksi? ____

(English translation)

<u>Planned interview for the fourth visit (or earlier if the patient is discharged)</u>

1. Time elapsed from the first treatment?

1-14 days/15-28 days/4-5weeks/6-8 weeks/More than 8 weeks

2. The intensity of pain during the past 24 hours?

No pain/Mild/Moderate/Severe/Unbearable

3. Present situation (compared to the 1st visit)?

Clearly better/maybe better/unchanged/maybe worse/definitely worse

4. If the patient was not treated four times… How many treatments were given?

1/2/3

5. Why was the patient discharged or not treated four times?

No treatment necessary-patient OK/Patient did not return/Referred (why?)
